# Deep learning to assess laryngoscope insertion depth during neonatal intubation with video laryngoscopy

**DOI:** 10.1038/s41372-025-02457-0

**Published:** 2025-10-27

**Authors:** Abrar Majeedi, Patrick J. Peebles, Yin Li, Ryan M. McAdams

**Affiliations:** 1 Department of Biostatistics and Medical Informatics, School of Medicine and Public Health, University of Wisconsin-Madison, Madison, WI USA; 2 Division of Neonatology, Department of Pediatrics, School of Medicine and Public Health, University of Wisconsin-Madison, Madison, WI USA; 3 Department of Computer Sciences, School of Computer, Data and Information Sciences, College of Letters and Science, University of Wisconsin-Madison, Madison, WI USA

**Keywords:** Medical imaging, Paediatrics

## Abstract

**Objective:**

Video laryngoscopy (VL) improves glottic visualization during neonatal intubation, but real-time guidance on blade insertion depth is lacking. We developed a deep learning model to classify insertion depth during VL as shallow, glottic zone, or deep.

**Study design:**

A deep learning model was trained on 298,955 annotated frames from 132 neonatal VL videos from two NICUs on one platform with fivefold cross-validation. 31 clinicians were surveyed regarding preferred device feedback modalities.

**Results:**

The model detected glottic zone and shallow insertion depths with F1 scores of 0.894 and 0.718, respectively. Deep insertion events were rare (2.7%) with low performance (F1 = 0.034). Most clinicians preferred visual, minimal prompts over voice or haptic feedback.

**Conclusion:**

AI-enabled VL may support blade-insertion depth assessment and training. Given the rarity of deep events, conclusions about deep insertion and clinical impact are limited. Future multi-site studies should focus on clinical integration and assessing outcomes.

## Introduction

Endotracheal intubation is a critical yet technically challenging procedure in neonatal care, particularly in preterm and low birthweight infants with anatomically distinct airways. Features such as a large occiput causing suboptimal head positioning, a proportionally larger tongue, and omega-shaped epiglottis can hinder glottic visualization [1]. First-pass success rates in neonatal intubation remain low, particularly among trainees, with some studies reporting success rates below 50% in neonatal intensive care units (NICUs) [2]. Repeated intubation attempts increase the risk of adverse events, including bradycardia, hypoxemia, and intraventricular hemorrhage [3, 4].

While many factors influence intubation success, including operator experience, infant size and age, premedication use, and device type, adequate visualization of the glottis is a fundamental requirement. Blade insertion depth is a direct determinant of whether the glottis is visible: advancing too little may obscure the view, while advancing too far can result in loss of landmarks. Because our dataset consisted of video laryngoscopy (VL) recordings, insertion depth provided a practical and clinically relevant focus for developing and testing automated guidance. VL has emerged as an important tool to address some of these challenges by providing indirect visualization of the airway via an external screen. Multiple studies have demonstrated that VL improves glottic visualization and first-pass success rates compared to direct laryngoscopy (DL) [5–7]. In neonates, VL has been associated with higher intubation success rates and fewer adverse events, such as oxygen desaturation [7–9]. However, despite these advantages, there are still opportunities to improve intubation outcomes using VL.

Artificial intelligence (AI), particularly computer vision methods using deep learning, is increasingly being applied to video data in neonatal intensive care units (NICUs) for diverse tasks [10–13], including neonatal video laryngoscopy [14]. AI offers a promising solution for augmenting VL in neonatal intubation. AI systems can enhance the interpretation of complex visual information, assist in video-derived anatomical landmark identification, and provide procedural guidance [15]. In the past few years, there has been a significant increase in research exploring AI applications in airway management, including glottic opening detection, phase recognition during intubation, and automated assessment of intubation quality [16, 17]. Despite these advances, relatively few studies have examined neonatal intubation using data derived from actual clinical practice; most existing work has focused on adult populations or simulation-based scenarios.

This study addresses this gap by developing a deep learning model capable of video-based detection of laryngoscope blade insertion depth during neonatal VL. Recent work has emphasized the importance of rigorous reporting standards and real-world applicability for AI in pediatric medicine [18]. In alignment with these emerging best practices, our approach integrates model transparency, clinician input, and usability assessment into the development of an AI-assisted intubation system. Building on prior work in glottic opening detection [14], the system classifies blade insertion depth as either too shallow or more optimally positioned near the glottis, enabling targeted feedback to support clinicians during intubation. In parallel, we surveyed neonatal providers to evaluate preferences for AI-guided feedback modalities, informing the design of future AI-augmented VL systems. By enabling frame-wise feedback on insertion depth, this approach aims to improve intubation efficiency, safety, and training in the NICU setting.

## Methods

### Data statistics and annotation

This study builds on a previously established dataset of 84 neonatal intubation recordings [14] and further expands the dataset to a total of 142 videos. These recordings were captured using a C-MAC video laryngoscope (Karl Storz, Tuttlingen, Germany) at two NICU facilities in Madison, Wisconsin: the Level III NICU at UnityPoint Health–Meriter Hospital from 2016 to 2024 and the Level IV NICU at the American Family Children’s Hospital from 2020 to 2024. Healthcare professionals performing the procedures were unaware of the study’s objective, ensuring that the recorded intubation practices remained natural and unbiased. Because videos were de-identified and not linked to patient identifiers, the number of unique infants cannot be reported. Some infants may have contributed more than one intubation video.

The videos were recorded at a resolution of 400 × 300 pixels with a frame rate of 30 *Hz*. To ensure high-quality data, all recordings underwent a manual review process to confirm the visibility of key anatomical structures and to verify that the airway was clearly visible without obstructions. After quality assessment, 132 videos (mean duration: 75.5 s) were retained for analysis, evenly split between the two NICU facilities (66 videos each). Videos were captured on the Karl Storz C-MAC platform. Under our IRB de-identification requirements, patient and operator identifiers were not retained, and clinical metadata were not linked to the videos.

To capture insertion depth transitions during intubation, each video was meticulously annotated by two neonatologists, who have been teaching and performing neonatal intubations for 7 and >15 years. The annotation protocol was developed collaboratively by jointly reviewing a subset of videos and agreeing on operational definitions for each insertion depth boundary. Each annotator independently labeled a separate half of the dataset; therefore, each video had a single rater, and we did not compute inter-rater reliability (e.g., Cohen’s κ or percent agreement). During routine labeling, annotators were not exposed to the other annotator’s labels. Ambiguous segments identified during labeling or subsequent review were then jointly reviewed and resolved by consensus. Annotators labeled from the videos alone and did not use automated model outputs. Annotations were performed using the VGG Video Annotator [19], ensuring a structured and consistent labeling process.

The annotators meticulously reviewed the intubation videos to track changes in insertion depth, specifically identifying when the video laryngoscope moved between shallow, glottic zone, and excessive depth. These insertion depth categories were defined based on the relationship of the laryngoscope to the trachea:

1. **Shallow**: The laryngoscope blade insertion depth was between the mouth opening and the glottis, with no visualization of the glottis or epiglottis on screen. The tongue was typically visible in this zone.
2. **Glottic Zone**: The laryngoscope blade insertion depth was in the region of the glottis, indicating proximity to the optimal intubation view. Views were classified as glottic zone only when glottic structures were visible, such as the vocal cords, arytenoid cartilages, epiglottis, or trachea. Frames showing only bubbles or indirect evidence of breathing without visualization of these structures were not classified as glottic zone.
3. **Deep**: The laryngoscope blade was advanced beyond the glottis and epiglottis. In this zone, the esophagus or other subglottic structures were typically visible on screen.

The primary objective was to capture transitions between distinct insertion depth categories, particularly between the shallow and glottic zones, as these were the most frequently occurring. Transitions were labeled only if they occurred at least one second apart. This approach helped reduce annotation density, ensuring that each labeled transition corresponded to a meaningful change in laryngoscope insertion depth, rather than to minor or transient movements.

Once transitions were annotated, each video frame was automatically categorized into one of the three insertion depth states—shallow, glottic zone, or deep—resulting in a total of 298,955 annotated frames, each representing a specific point in the intubation process. Because deep frames were relatively scarce, the study primarily focuses on the shallow and glottic zones, which were more commonly encountered (Table 1). This study was based on a convenience sample of all available neonatal VL recordings at our institution that met inclusion criteria during the study period. As such, sample size was determined by data availability rather than by a priori power calculation.

**Table 1:** Data annotation statistics. (source: authors’ data).

Insertion depth	Number of annotated frames	Average number of frames per video	Average duration per video (sec)
Shallow	74300	542.42	18.08
Glottic Zone	216553	1640.55	54.68
Deep	8102	61.38	2.04
*All*	*298,955*	*2264.81*	*75.49*

### Online insertion depth detection with deep learning

To classify insertion depth, we employed an Inflated 3D Convolutional Network (I3D) [20] pretrained on the Kinetics dataset [20], with a multi-layer perceptron (MLP) head for classification. I3D is well-suited for spatiotemporal modeling in video data leveraging 3D convolutions to capture both spatial and temporal dependencies. Its ability to model temporal relationships between consecutive frames makes it particularly effective for dynamic tasks such as insertion depth detection. While I3D supports both optical flow and RGB inputs, we used only the RGB stream to improve computational efficiency.

Videos were down-sampled and processed at 10 Hz to reduce temporal redundancy, enhance computational throughput, and minimize visual clutter. Since human actions occur slowly relative to this sampling rate, 10 fps preserves sufficient temporal resolution for accurate insertion depth detection while substantially reducing computational cost. The model operated on 8-frame clips (0.8 s at 10 Hz), providing temporal context from the preceding seven frames to classify the final frame of each clip as representing either the shallow or glottic zone insertion depth.

**Fig. 1:**
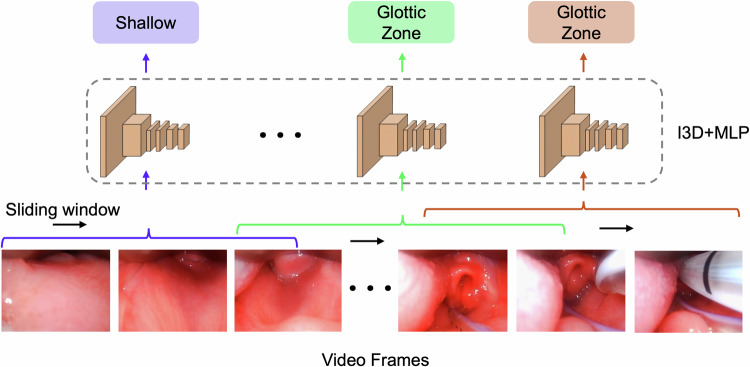
Overview of video-based insertion depth prediction in neonatal video laryngoscopy using deep learning. The system processes video laryngoscopy frames in a sequential, frame-by-frame manner to classify blade insertion depth as shallow or within the glottic zone.

The I3D backbone extracted a 1024-dimensional feature vector from each clip, which was then passed to the MLP head comprising three linear layers interleaved with ReLU activations and a dropout layer (rate = 0.1) to mitigate overfitting. During training, clips were sampled without overlap to increase data diversity and reduce correlation between samples. During evaluation, an overlapping sliding-window approach was used so that predictions were generated for every frame, preserving temporal continuity and enabling a comprehensive assessment of performance. An overview of this pipeline is shown in Fig. 1.

Model training and evaluation were conducted using fivefold cross-validation to ensure robust performance assessment. Data splits were made at the video/encounter level, so that all frames from a given video remained within the same fold. This prevented frame-level leakage between training and validation sets. In most cases, each video corresponded to a distinct patient; however, because the dataset was de-identified, we could not determine if some infants contributed more than one video. This approach accounts for differences in sample complexity and potential site-specific effects, reducing the risk of overfitting.

End-to-end training was performed using the AdamW optimizer [21] with learning rates of 5 × 10⁻⁴ for the MLP head and 5 × 10⁻⁵ for the I3D backbone and a weight decay of ${10}^{-3}$. The model was trained for 60 epochs with a batch size of 64. In line with common practice, the model from the final epoch was chosen for testing and inference. Focal loss [22] (with parameters: $\alpha =0.25,\gamma =2.0$) was used to address class imbalance. During training, frames were randomly cropped to 300×300 pixels; during testing, center cropping of the same size was applied. The experiments were conducted on two NVIDIA T4 GPUs (16 GB each), utilizing approximately 14.5 GB of memory per GPU. The processing throughput was approximately 26 clips per second, with each clip consisting of 8 frames.

To improve temporal consistency and reduce transient mispredictions, we applied a post-processing step using majority voting within a five-frame sliding window. The most frequent label across the current and four preceding predictions was selected, smoothing the output with minimal computational overhead.

### Participant survey

To systematically assess clinician preferences for AI guidance during neonatal intubation, we designed a structured survey. The survey specifically targeted clinicians routinely performing neonatal intubations, yet with varying experience levels, including neonatologists, fellows, and nurse practitioners, and a NICU hospitalist from the Level IV NICU at the American Family Children’s Hospital in Madison, Wisconsin, United States of America. The survey was sent electronically to 48 clinicians of whom 31 completed it, resulting in a 65% response rate.

At the outset, participants were first asked to indicate their preferred modes of AI feedback during intubation: voice prompts, text prompts, visual indicators, or haptic feedback. This established general modality preferences for video derived AI support.

Subsequent survey sections explored each modality in greater detail. For voice prompts, participants rated the importance of features such as volume control, speech speed, voice type (male, female, or neutral), muting capability, and the use of concise, standardized phrases. For visual feedback, preferences were assessed for text size, color-coded cues (e.g., red, yellow, or blue), screen positioning, brevity and standardization of text, and the ability to quickly dismiss prompts. Participants also evaluated additional visual elements, including directional arrows for corrective actions, anatomical highlighting, color-coded guidance schemes, and minimalistic icons representing next steps.

Participants were also asked to identify concerns associated with live AI guidance, including cognitive overload, procedural distraction, interference with team communication, and the risk of misinterpreting AI outputs. Finally, the survey queried preferences for the timing of feedback delivery: continuous feedback, adjustment-only feedback, or feedback available upon request.

This structured design enabled us to capture both high-level modality preferences and detailed user interface considerations essential for developing effective AI-guided systems in neonatal intubation.

## Results

### Insertion depth detection model performance

**Fig. 2:**
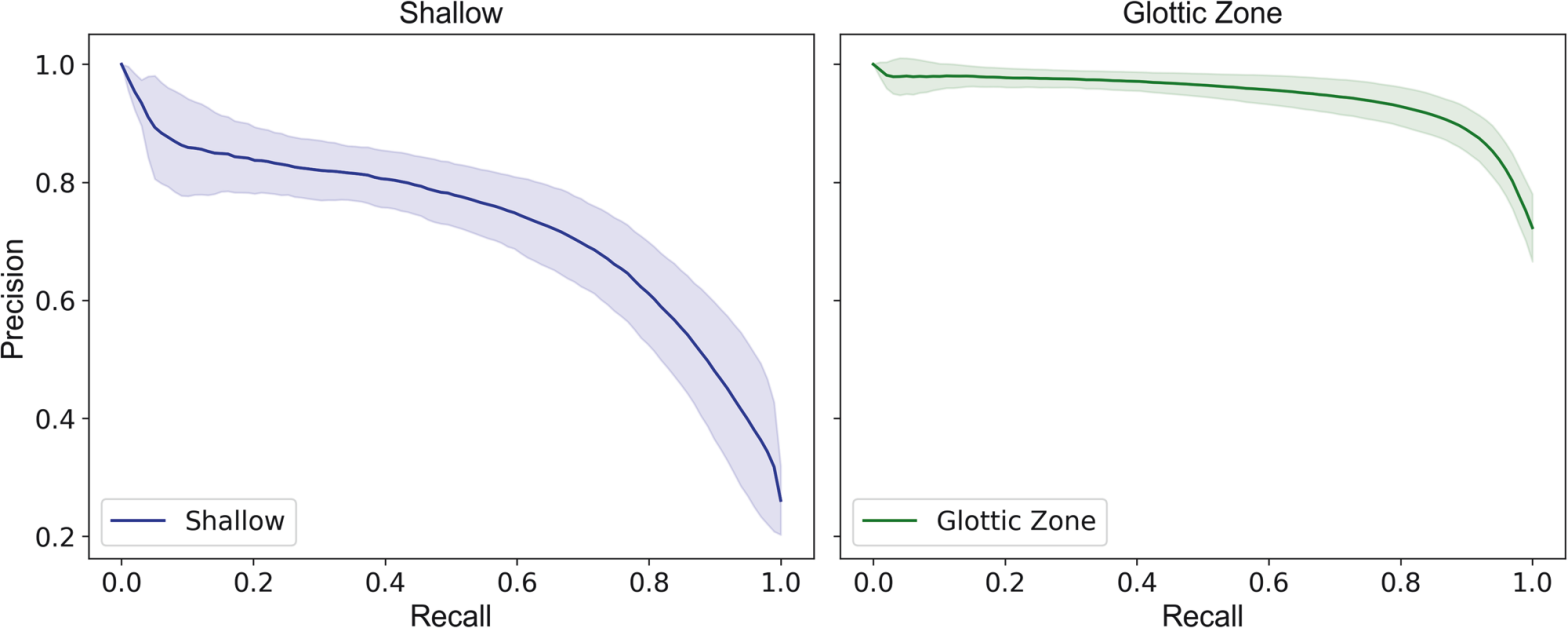
Precision–recall curves for insertion depth classification. The left panel shows the precision–recall curve for detection of the shallow insertion depth, and the right panel shows the curve for detection of the glottic zone. (Source: Authors’ data).

The model was trained on 298,955 annotated frames (24.9% shallow, 72.4% glottic zone and 2.7% deep) from 132 neonatal intubation videos. Based on fivefold cross-validation (*n* = 5), the model achieved an F1 score of 0.894 ± 0.016 (95% CI: 0.874, 0.914) for the glottic zone insertion depth, with a precision of 0.902 ± 0.035 (95% CI: 0.859, 0.945) and a recall of 0.892 ± 0.025 (95% CI: 0.861, 0.923). For the shallow insertion depth, the F1 score was 0.718 ± 0.041 (95% CI: 0.667, 0.769), with a precision of 0.676 ± 0.070 (95% CI: 0.589, 0.763) and a recall of 0.764 ± 0.032 (95% CI: 0.724, 0.804). Performance metrics for the deep insertion depth were substantially lower, with an F1 score of 0.034 ± 0.036 (95% CI: 0.000, 0.078), precision of 0.072 ± 0.088 (95% CI: 0.000, 0.182), and recall of 0.024 ± 0.026 (95% CI: 0.00, 0.056) as shown in Table 2. Precision-recall curves for the shallow and glottic insertion depths are shown in Fig. 2.

**Table 2:** Insertion depth detection results.

Insertion Depth	F1 score	Precision	Recall
Shallow	0.718$\pm$ 0.041 [0.667, 0.769]	0.676$\pm$ 0.070 [0.589, 0.763]	0.764$\pm$ 0.032 [0.724, 0.804]
Glottic Zone	0.894$\pm$ 0.016 [0.874, 0.914]	0.902$\pm$0.035 [0.859, 0.945]	0.892$\pm$ 0.025 [0.861, 0.923]
Deep	0.034$\pm$ 0.035 [0.000, 0.078]	0.072$\pm$ 0.088 [0.000, 0.182]	0.024$\pm$ 0.026 [0.00, 0.056]

F1 score, precision and recall are reported for each insertion depth category. The metrics are averaged over five cross-validation folds, with the mean ± standard deviation and 95% confidence interval provided in parentheses. (Source: Authors’ data).

Post-processing with a five-frame sliding window using majority voting reduced transient mispredictions and improved the temporal consistency of insertion depth classifications. Quantitative results of the post-processing impact are available in the supplementary materials.

### Integration with glottic opening detection

**Fig. 3:**
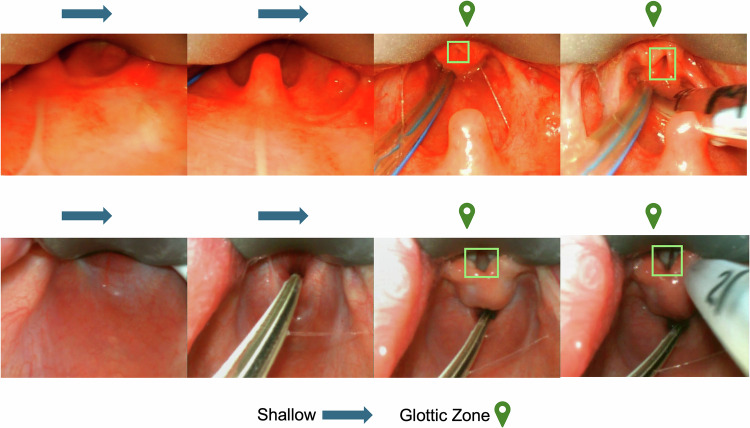
Example of AI-generated visual guidance during neonatal video laryngoscopy. (Source: Authors’ Data).

The model was integrated with a previously developed glottic opening detection method [14]. Glottic opening detection, aiming to recognize and localize glottic opening in an input video, was conditionally activated only when the predicted insertion depth was classified as within the glottic zone. Frame-level visualizations of this integrated output are presented in Fig. 3.

### Participant survey results

**Fig. 4:**
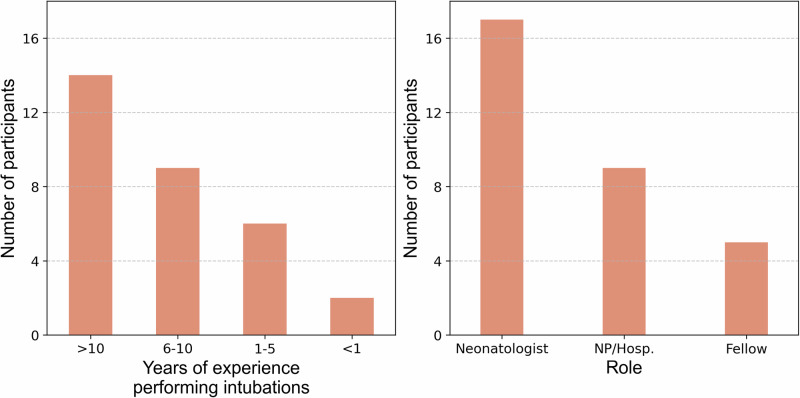
Survey participant characteristics. The left panel shows the distribution of participants by years of neonatal intubation experience, and the right panel shows distribution by professional role. Respondents included neonatologists, fellows, and nurse practitioners/NICU hospitalist (NP/Hosp) from a Level IV NICU. (Source: Authors’ data).

Thirty-one participants consisting of neonatologists (*n* = 17), fellows (*n* = 5), neonatal nurse practitioners (NP; *n* = 8) and a NICU hospitalist (*n* = 1) from a Level IV NICU participated in the feedback survey. A summary of participants by clinical role and years of intubation experience is shown in Fig. 4.

**Fig. 5:**
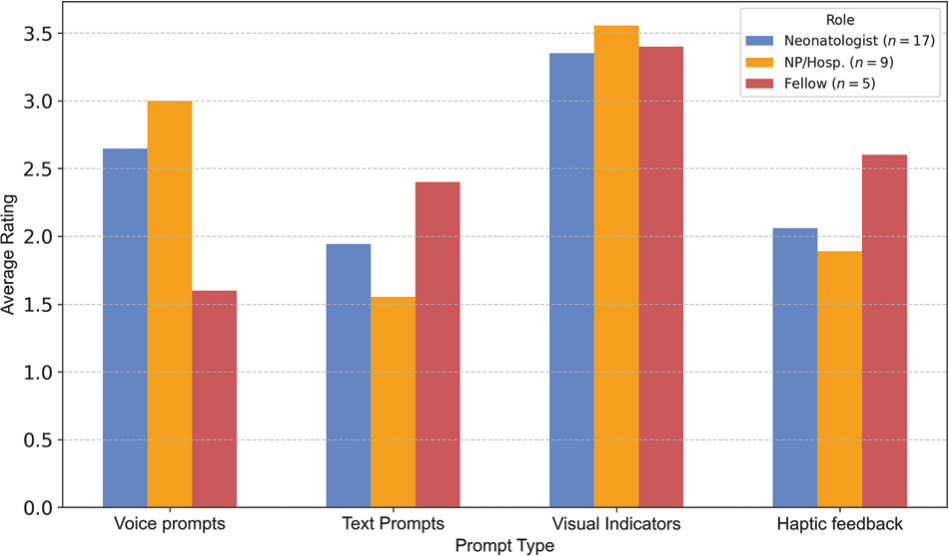
Clinician preferences (on a 5-point Likert scale) for AI guidance modalities during neonatal video laryngoscopy. Participants (neonatologists, *n* = 17; fellows, *n* = 5; and neonatal nurse practitioners/NICU hospitalist (NP/Hosp.), *n* = 9; rated four feedback modalities—voice prompts, text prompts, visual indicators, and haptic feedback—for use during neonatal intubation. Ratings reflect preferences across all clinician roles. (Source: Authors’ data).

Across all roles, visual feedback was rated as the most preferred modality on a 5-point Likert scale (1 = least preferred, 5 = most preferred), with mean ratings of 3.56 (median: 4, IQR: [3,4]) for NP/Hosp. group, 3.40 (median: 4, IQR: [3,4]) for fellows, and 3.35 (median: 4, IQR: [3,4]) for neonatologists. Voice prompts and text prompts were rated lower, while haptic feedback received the lowest preference scores. These trends are illustrated in Fig. 5, with detailed results provided in Supplementary Table 1. Within the voice prompt category, participants gave the highest average ratings to concise, standardized phrases (>4.2), followed by volume control (≈3.2–3.8). Voice type (male, female, or neutral) was consistently rated as less important (Supplementary Table 2).

For text and visual prompts, participants preferred adjustable screen positioning and the ability to dismiss prompts quickly. Ratings for other features—such as color-coded schemes, directional arrows, and anatomical highlighting—are detailed in Supplementary Tables 3, 4.

Regarding concerns, participants most frequently cited distraction during procedures and the risk of misinterpreting guidance. Cognitive overload and interference with team communication were reported less frequently. These concerns varied slightly by modality and clinician role, as shown in Supplementary Tables 5–8. Preferences for feedback timing are summarized in Supplementary Table 9. Most participants preferred feedback to be displayed only when an adjustment was needed, rather than continuously or upon request.

## Discussion

In this study, we developed and evaluated a deep learning model for classification of laryngoscope insertion depth during neonatal VL intubation. Trained on over 298,000 annotated video frames from 132 intubation recordings, the model demonstrated strong performance in distinguishing shallow from glottic-zone insertion depths, demonstrating F1 scores of 0.718 and 0.894, respectively. The system was designed to operate in an online fashion, producing causal predictions based only on past and present frames, thereby supporting the possibility of real-time clinical deployment. By contrast, performance for detecting deep insertion was poor (F1 = 0.034), reflecting the rarity of these events in the dataset. Together, these results provide proof of concept for AI-enabled feedback on insertion depth, highlighting both promise and current limitations.

Our findings are consistent with and extend prior research applying AI to airway management. In prior work, we demonstrated the feasibility of using YOLOv8 [23], a deep learning object detection model, to identify the glottic opening in neonatal VL videos with a precision of 80.8% and recall of 75.3% [14]. Their system detected the glottic opening 0.31 s faster than human providers, highlighting the potential of AI to serve as a procedural decision-support tool. Similarly, Wu et al. [16] applied YOLOv3 [24] for time-sequence analysis of tracheal intubation in adults, achieving high accuracy in anatomical landmark recognition and procedural phase classification [16, 25], while other groups have applied AI to glottic detection, airway anatomy recognition, and automated assessment of intubation quality [15]. These efforts highlight growing momentum in the field of AI-assisted airway management. Our contribution differs by focusing on blade insertion depth, which directly influences whether the glottis is visualized and intubation is possible. By detecting suboptimal depth, the model addresses a critical procedural step that is within the operator’s control and amenable to corrective adjustment.

Clinically, neonatal intubation success has been improved through structured supervision, simulation-based training, and standardized premedication [2–4], as well as through widespread adoption of VL [5–9] .Each of these strategies addresses important contributors to procedural difficulty, including operator experience, infant physiology, and device type. Our model complements these approaches by focusing on insertion depth, a modifiable factor that determines glottic visualization. By signaling when the blade is advanced insufficiently or excessively, AI-enabled feedback could reduce failed attempts, shorten procedure time, and decrease complications such as desaturation, bradycardia, or airway trauma. Importantly, while trainees may derive the greatest benefit, experienced providers may prefer minimal intervention. Designing systems that allow adjustable feedback levels or user-controlled modes will be essential for broader clinical adoption.

Despite its strengths, several limitations warrant discussion. The dataset was collected at two NICUs in the same metropolitan area using a single VL platform (Karl Storz C-MAC). This uniformity reduces variability but may also limit generalizability to other devices, practice settings, or video characteristics. Differences in hardware (lens geometry, color rendering, sensor properties), resolution, compression, or lighting (ambient or on-scope) could alter input distributions and degrade performance. Furthermore, the videos were de-identified and not linked to clinical records, preventing stratification by infant gestational age, birthweight, indication for intubation, pathology, or operator level of training, all factors that influence intubation success. We also could not assess the impact of premedications, including neuromuscular blockade, which affect airway conditions and ease of intubation.

Another limitation relates to annotation. Each video was singly labeled, so we could not compute inter-rater reliability (e.g., Cohen’s κ). While annotators conducted initial calibration on a subset of videos and resolved ambiguities by consensus, dual labeling of a stratified subset with formal agreement metrics would strengthen reliability in future datasets. Our operational definition of the “glottic zone” also warrants clarification: it encompassed any view where glottic structures (e.g., vocal cords, arytenoids, epiglottis, or trachea) were visible, rather than a narrower “optimal vocal cord” view. While this broader definition supports real-time guidance, finer-grained classification may ultimately be necessary for advanced clinical applications. Finally, the evaluation was retrospective. Prospective, multi-site validation studies in clinical workflows that include EHR linkage and multiple VL platforms will be needed to assess effects on intubation success, procedure duration, and patient outcomes, as well as to quantify generalizability and support device-specific calibration.

Deep insertion deserves specific emphasis as a clinically important error state. Excessive advancement of the blade can obscure the glottis, cause esophageal intubation, or increase the risk of airway trauma. In our dataset, deep frames accounted for only 2.7% of all frames and were often brief. Temporal smoothing, while helpful in reducing noise, may have muted some of these episodes, contributing to poor performance. Strategies to improve recognition of deep insertion include enriching training data with additional examples, targeted review of existing archives, multi-site data collection, rebalancing loss functions, incorporating difficult examples, modifying temporal decision rules to allow rapid early warnings, and adopting operating points that favor sensitivity for deep events. Future work should report class-specific metrics and calibrated probabilities to better characterize model performance in this error state.

Beyond intubation, AI-based video analysis has the potential to be adapted for other complex procedures that rely on video-based image interpretation and precise hand-eye coordination. Similar systems could be developed to assist with bronchoscopy, endoscopy, or point-of-care ultrasound, providing dynamic feedback and serving as digital mentors for less experienced providers. For intubation specifically, future systems could progress from simple classification of depth to actionable guidance, recommending maneuvers such as slight withdrawal of the blade or adjustment of lateral positioning. Integration with existing VL platforms could enable visual overlays without disrupting procedural flow or requiring additional hardware, supporting unobtrusive human–AI collaboration in high-acuity environments such as the NICU. These technologies can serve as digital mentors, bridging the gap between novice skill levels and expert proficiency in high-stakes clinical environments. This AI digital enhancement may be particularly beneficial in lower resource and rural settings where the on-screen feedback can guide a less experienced operator through a life-saving procedure.

However, it is important to emphasize that the current model is not intended for clinical use and has not undergone regulatory review or approval. This work represents an early proof of concept, and future deployment will require rigorous prospective validation, usability testing, and formal regulatory clearance. While this model serves as an initial step, future systems, if carefully developed and validated, may ultimately support safer and more effective neonatal intubation, particularly for less experienced providers.

## Conclusion

In summary, this study demonstrates the feasibility of AI-assisted detection of insertion depth during neonatal VL intubation. While current performance is limited for rare deep insertion events, the model shows strong accuracy for shallow and glottic depths, highlighting potential value for real-time feedback and training. Addressing class imbalance, expanding datasets across diverse populations and devices, and conducting prospective validation will be critical next steps. With rigorous development, regulatory approval, and careful integration into clinical practice, AI-enabled feedback systems may contribute to safer and more effective neonatal intubation and serve as a foundation for broader applications in procedural medicine.

## Supplementary information

Supplement

## Data Availability

The data collected for this study, including individual patient videos and the corresponding data dictionary, will not be made publicly available due to HIPAA concerns and institutional policies. The videos were originally recorded for internal quality improvement purposes, and there is no current approval from the hospital’s Institutional Review Board to share these sensitive data publicly. Ensuring patient privacy and confidentiality is a priority, and as of now, the use of these videos is restricted to authorized personnel within our institution.

## Code availability

The computer code used for this study is available upon request from the corresponding author.
