# Supplementary material for: Deep learning to assess laryngoscope insertion depth during neonatal intubation with video laryngoscopy
Source: J Perinatol. 2025 Oct 27;46(1):43–9. doi: 10.1038/s41372-025-02457-0 (PMC12815674; doi:10.1038/s41372-025-02457-0)
Supplement: Supplementary file 1 — Supplement [file 41372_2025_2457_MOESM1_ESM.pdf]

Supplementary materials

Role	Voice Prompts	Text Prompts on Screen	Visual Indicators / Icons	Haptic Feedback
Fellow (n=5)	1.60, 1.0 [1.0–1.0]	2.40, 2.0 [2.0–3.0]	3.40, 4.0 [3.0–4.0]	2.60, 3.0 [2.0–3.0]
NP/Hosp. (n=9)	3.00, 3.0 [2.0–4.0]	1.56, 1.0 [1.0–2.0]	3.56, 4.0 [3.0–4.0]	1.89, 2.0 [1.0–3.0]
Neonatologist (n=17)	2.65, 3.0 [1.0–4.0]	1.94, 2.0 [1.0–3.0]	3.35, 4.0 [3.0–4.0]	2.06, 2.0 [2.0–2.0]

Supplementary Table 1. Preference for AI guidance via voice prompts. Values represent the weighted mean, median, and interquartile range [IQR] *(Source: Authors' data)*

Role	Volume Control	Speed of Speech	Voice type (Male/Female /Neutral)	Ability to Mute Quickly	Concise, Standardize d Phrases
Fellow (n=5)	3.80, 4.0 [3.0–4.0]	3.40, 4.0 [3.0–4.0]	1.40, 1.0 [1.0–1.0]	2.20, 2.0 [2.0–2.0]	4.20, 5.0 [5.0–5.0]
NP/Hosp. (n=9)	3.56, 4.0 [3.0–4.0]	3.00, 3.0 [2.0–4.0]	1.22, 1.0 [1.0–1.0]	2.78, 3.0 [2.0–3.0]	4.44, 5.0 [4.0–5.0]
Neonatologist (n=17)	3.19, 3.0 [3.0–4.0]	2.94, 3.0 [2.0–4.0]	1.38, 1.0 [1.0–2.0]	3.00, 2.0 [2.0–4.2]	4.50, 5.0 [4.0–5.0]

Supplementary Table 2. Preference for AI guidance via voice prompts. Values represent the weighted mean, median, and interquartile range [IQR] **(***Source: Authors' data)*

Role	Text size	Color-coded guidance (e.g., red/yellow/blue)	Position on screen	Concise, standardized phrases	Ability to dismiss quickly
Fellow (n=5)	3.50, 3.5 [2.8–4.2]	2.00, 1.5 [1.0–2.5]	4.50, 5.0 [4.5–5.0]	3.00, 3.0 [2.8–3.2]	2.00, 1.5 [1.0–2.5]
NP/Hosp. (n=9)	2.67, 2.0 [2.0–4.0]	2.44, 3.0 [2.0–3.0]	4.33, 5.0 [4.0–5.0]	3.89, 4.0 [3.0–4.0]	1.67, 1.0 [1.0–2.0]
Neonatologist (n=17)	3.41, 4.0 [2.0–4.0]	2.06, 2.0 [1.0–3.0]	3.76, 4.0 [3.0–5.0]	3.12, 3.0 [2.0–3.0]	2.65, 3.0 [1.0–4.0]

Supplementary Table 3. Preference for AI guidance via text prompts. Values represent the weighted mean, median, and interquartile range [IQR] *(Source: Authors' data)*

Role	Directional arrows showing adjustment direction	Highlighting anatomical structures	Color-coded guidance (e.g., red/yellow/green)	Simple icons representing actions
Fellow (n=5)	3.40, 4.0 [3.0–4.0]	3.40, 3.0 [3.0–4.0]	1.60, 1.0 [1.0–2.0]	1.60, 2.0 [1.0–2.0]
NP/Hosp. (n=9)	3.29, 3.0 [3.0–4.0]	3.00, 3.0 [2.5–3.5]	1.57, 1.0 [1.0–1.5]	2.14, 2.0 [1.5–2.5]
Neonatologist (n=17)	2.81, 3.0 [2.0–4.0]	3.50, 4.0 [3.0–4.0]	1.44, 1.0 [1.0–2.0]	2.25, 2.5 [1.0–3.0]

Supplementary Table 4. Preference for AI guidance via Visual Indicators prompts. Values represent the weighted mean, median, and interquartile range [IQR] *(Source: Authors' data)*

Voice prompt concerns	Fellow (n=5)	NP/Hosp. (n=9)	Neonatologist (n=17)
Cognitive overload	0	0	0.18
Distraction during procedure	0.2	0.22	0.24
Interference with team communication	0.6	0.56	0.41
Misinterpretation of guidance	0.2	0	0.06
Not concerned about any of these issues	0	0.22	0.12

Supplementary Table 5. Voice prompt concerns. Values represent the proportion of providers within each role who reported the corresponding concern. *(Source: Authors' data)*

Text prompt concerns	Fellow (n=5)	NP/Hosp. (n=9)	Neonatologist (n=17)
Cognitive overload	0.2	0.22	0.13
Distraction during procedure	0.8	0.56	0.75
Interference with team communication	0	0	0
Misinterpretation of guidance	0	0	0.12
Not concerned about any of these issues	0	0.22	0

Supplementary Table 6. Text prompt concerns. Values represent the proportion of providers within each role who reported the corresponding concern. *(Source: Authors' data)*

Visual Indicator Concerns	Fellow (n=5)	NP/Hosp. (n=9)	Neonatologist (n=17)
Cognitive overload	0.2	0	0.12
Distraction during procedure	0.4	0.22	0.41
Interference with team communication	0.0	0.0	0.0
Misinterpretation of guidance	0.2	0.44	0.29
Not concerned about any of these issues	0.2	0.33	0.18

Supplementary Table 7. Visual Indicator Concerns. Values represent the proportion of providers within each role who reported the corresponding concern. *(Source: Authors' data)*

Haptic feedback Concern	Fellow (n=5)	NP/Hosp. (n=9)	Neonatologist (n=17)
Cognitive overload	0.2	0.44	0.24
Distraction during procedure	0.4	0.22	0.35
Interference with team communication	0.0	0.0	0.0
Misinterpretation of guidance	0	0.33	0.35
Not concerned about any of these issues	0.4	0	0.06

Supplementary Table 8. Haptic Feedback concerns. Values represent the proportion of providers within each role who reported the corresponding concern. *(Source: Authors' data)*

Feedback frequency	Fellow (n=5)	NP/Hosp. (n=9)	Neonatologist (n=17)
Continuous feedback	0.2	0.22	0.06
Only when adjustment needed	0.6	0.56	0.65
Only when specifically requested	0.2	0.22	0.29

Supplementary Table 9. Feedback frequency. Values represent the proportion of providers within each role who preferred the corresponding feedback frequency. *(Source: Authors' data)*
